# Everolimus Treatment for Chronic Lung Allograft Dysfunction in Lung Transplantation

**DOI:** 10.3390/life14050603

**Published:** 2024-05-08

**Authors:** David Iturbe-Fernández, Alicia de Pablo Gafas, Víctor Manuel Mora Cuesta, Rodrigo Alonso Moralejo, Carlos Andrés Quezada Loaiza, Virginia Pérez González, Daniel López-Padilla, José M. Cifrián

**Affiliations:** 1 Lung Transplant Unit, Pulmonary Medicine Department, Marqués de Valdecilla University Hospital, 39008 Santander, Spain; victormanuel.mora@scsalud.es (V.M.M.C.); josemanuel.cifrian@scsalud.es (J.M.C.); 2 Lung Transplant Unit, Pulmonary Medicine Department, Doce de Octubre University Hospital, 28041 Madrid, Spain; alic1575@separ.es (A.d.P.G.); ralonsomoralejo@gmail.com (R.A.M.); andresquezadal@gmail.com (C.A.Q.L.); vluz71@hotmail.com (V.P.G.); 3 CIBER Respiratory Diseases (CIBERES), Carlos III Health Institute, 28029 Madrid, Spain; 4 Pulmonary Medicine Department, Gregorio Marañón University Hospital, 28007 Madrid, Spain

**Keywords:** lung transplantation, chronic lung allograft dysfunction, everolimus

## Abstract

Our study aims to evaluate the effect of everolimus treatment on lung function in lung transplant (LT) patients with established chronic lung allograft dysfunction (CLAD). Methods: This retrospective study included LT patients in two reference LT units who started everolimus therapy to treat CLAD from October 2008 to October 2016. We assessed the variation in the maximum forced expiratory volume in the first second (FEV_1_) before and after the treatment. Results: Fifty-seven patients were included in this study. The variation in the FEV_1_ was −102.7 (149.6) mL/month before starting everolimus compared to −44.7 (109.6) mL/month within the first three months, +1.4 (63.5) mL/month until the sixth month, and −7.4 (46.2) mL/month until the twelfth month (*p* < 0.05). Glomerular filtrate remained unchanged after everolimus treatment [59.1 (17.5) mL/min per 1.73 m^2^ at baseline and 60.9 (19.6) mL/min per 1.73 m^2^, 57.7 (20.5) mL/min per 1.73 m^2^, and 57.3 (17.8) mL/min per 1.73 m^2^, at 1, 3, and 6 months, respectively] (*p* > 0.05). Everolimus was withdrawn in 22 (38.6%) patients. The median time to withdrawal was 14.1 (5.5–25.1) months. Conclusions: This study showed an improvement in FEV_1_ decline in patients with CLAD treated with everolimus. However, the drug was withdrawn in a high proportion of patients.

## 1. Introduction

Chronic lung allograft dysfunction (CLAD) is a persistent decline in lung function that affects 48% of lung transplant (LT) recipients in the first five years post transplantation [1]. Classically, CLAD has been synonymous with bronchiolitis obliterans syndrome (BOS), which is defined by an obstructive ventilatory pattern. However, restrictive allograft syndrome (RAS) has increasingly been described as a different phenotype of CLAD characterized by a restrictive ventilatory condition [2]. Although multiple therapeutic strategies have been employed to prevent or treat CLAD, none have been significantly successful [3,4,5,6,7,8,9]. Hence, it remains the leading cause limiting LT survival. Therefore, it is crucial to investigate the efficacy of CLAD therapies to establish treatment recommendations for the main condition that limits long-term survival in LT patients. Everolimus is an inhibitor of mammalian target of rapamycin (imTOR). It exerts immunosuppressive activity by binding to the cytosolic FK506 binding protein 12 complex, blocking the activity of the serine–threonine kinase mTOR. Thus, it inhibits kinases responsible for the late gene transcription of multiple cytokines, including IL-2, ultimately restraining the proliferation of lymphocytes and fibroblasts and the expression of proliferative cytokines [10,11]. In LT, it has been evaluated for preventing CLAD and renal dysfunction, both as a replacement for cell cycle inhibitors [12,13] or as an addition to standard treatment as part of quadruple therapy [14,15,16,17]. In other solid organ transplants, imTORs have been recommended in recurrent cytomegalovirus (CMV) infections or malignancy. Based on the first published experiences regarding the use of everolimus in lung and other solid organ transplantations and its effect on lung function in patients diagnosed with CLAD, a Spanish consensus on indications and management was achieved [18,19,20,21].

Bearing in mind that CLAD is a fibrotic process which is unlikely to be reversible [22,23], the objective of treatment is to achieve a slowdown in the rate of the loss of lung function, which would mean a delay in the functional deterioration of the graft. Therefore, our study aimed to evaluate the effect of everolimus treatment on lung function in LT patients with established CLAD.

## 2. Materials and Methods

### 2.1. Population

This is a retrospective observational study comprising a cohort of LT patients who started everolimus treatment in two reference centers in Spain from October 2008 to October 2016. Follow-up concluded in February 2023. This study was conducted in accordance with the Declaration of Helsinki, and its wording is in line with the STROBE Initiative, having been approved by the local Ethics and Clinical Research Committee (resolution number 15/261).

### 2.2. Study Variables

The following variables were documented: demographic characteristics, the primary diagnosis that motivated the transplantation, the type of transplant (unilateral or bilateral), the immunosuppressive scheme implemented before the everolimus treatment, the date of everolimus onset, and if it existed in addition to CLAD, another reason that justified the indication for everolimus. Regarding drug-related toxicity, we assessed the effect on the renal function (serum creatinine and glomerular filtrate (GF), calculated using the Modification of Diet in Renal Disease (MDRD) equation [24]); the lipid profile (total cholesterol and triglyceride levels); and in the case of discontinuation of the medication, the time to withdrawal and its cause.

Lung function was evaluated using the FEV_1_ at months 6 and 3 before the start of everolimus, at the onset of everolimus (day “0”), and at months 1, 3, 6, and 12 after its introduction. The primary outcome was the variation in the FEV_1_ before and after everolimus treatment in order to evaluate its effect on lung function decline. The slope of the fall or gain in lung function (P) was calculated in milliliters per month (mL/month) as follows:

P(month *x* to *y*) = (FEV_1_ month *y* − FEV_1_ month *x*)/(*y* − *x*)

CLAD was classified into bronchiolitis obliterans syndrome (BOS) and restrictive allograft syndrome (RAS) according to current recommendations [2]. BOS was graduated as recommended by the 2001 consensus [22].

Regarding pharmacology, the following variables were collected: serum levels of calcineurin inhibitor (CNI) in the months prior to the onset of the imTOR and at the first, third, and sixth months thereafter, the percentage of patients who maintained antimetabolites after the initiation of everolimus, and serum everolimus trough levels at the first, third, sixth, and twelfth months, measured in nanograms per milliliter (ng/mL).

Monitoring ended in February 2023. Thus, all the patients included were followed up with for at least 5 years. The date and cause of death were documented. The data were obtained from the clinical records and databases of both lung transplantation units.

### 2.3. Protocol and Monitoring

In both centers, the immunosuppression protocol consists of a combination of a CNI (with tacrolimus as the first option), antimetabolites (with mycophenolate mofetil (MMF) or mycophenolic acid (MPA) as the first option), corticosteroids, and basiliximab induction therapy. Azithromycin is initiated four weeks after the transplant for all the patients in one center. In the other center, until January 2017, it was indicated if CLAD was diagnosed and, after January 2017, it was indicated in all the patients four weeks after the procedure. Everolimus was started at the discretion of the clinician responsible for the patient. The same scheme was followed in all cases: on day zero, it was started at doses between 0.75 and 1 mg twice daily. Serum levels were assessed every 3 to 5 days. When the therapeutic range of everolimus (5–8 ng/mL) was reached, tacrolimus or cyclosporine was lowered in order to reduce its trough level to 50% of that prior to the introduction of everolimus. The corticosteroid dose was unchanged. The decision to continue or not with a fourth line of immunosuppression (antimetabolites) was left to the discretion of the clinician responsible for the patient.

### 2.4. Statistics

Qualitative variables are presented as absolute numbers and percentages, while quantitative variables are presented as means (standard deviation) or medians (interquartile range), as the case may be. The normal distribution of variables was evaluated using the Kolmogorov–Smirnov test. For the purposes of the main objective of this study, the FEV_1_ slopes from before and after everolimus were compared, considering them continuous quantitative variables. Differences between groups for continuous variables were compared univariately using Student’s *t*-test when the variable had a normal distribution or the Mann–Whitney U test when it was not normal. Differences in quantitative variables in paired samples were evaluated using Student’s *t*-test for paired samples and with the Wilcoxon range under the conditions of normality and a lack of normality, respectively. A repeated-measures ANOVA was used to evaluate differences across three or more measures of the same variable, and Bonferroni’s post hoc test was conducted to explore specific differences between groups. A survival analysis was performed using the Kaplan–Meier method, and the differences in survival outcomes between groups were compared using a log-rank test.

A *p*-value of less than 0.05 was considered statistically significant. Statistical analyses were performed using IBM SPSS V.26 (IBM SPSS Statistics, Armonk, NY, USA).

## 3. Results

During the study period, everolimus was initiated in 101 patients. In 57 (56.3%), CLAD was the primary reason for starting this therapy, so they were included in this study. In addition to CLAD, nine patients had renal insufficiency, and one patient had neoplasms. The remaining non-CLAD everolimus indications were renal insufficiency, repeated CMV infections, and malignancy.

Among the 57 patients with CLAD in which everolimus was initiated, 38 (67%) were male, and the median age at the time of transplantation was 55.8 (51.2–61.5) years. Underlying lung diseases were as follows: chronic obstructive pulmonary disease (n: 29, 51%), interstitial lung disease (n: 17, 30%), pulmonary arterial hypertension (n: 5, 9%), and other conditions (n: 6, 10%). The median time from transplantation to the onset of everolimus was 27.1 (16–38.8) months, and the patients analyzed received everolimus for a median of 21.6 (6.1–73.7) months. The demographic and clinical data of these patients are summarized in Table 1.

Regarding the type and severity of CLAD, the baseline BOS was stage 0-p in 18 (31.6%), stage 1 in 14 patients (24.6%), stage 2 in 13 patients (22.8%), and stage 3 in 6 patients (10.5%). Three patients (5%) met the criteria for RAS prior to everolimus therapy.

### 3.1. Pulmonary Function

**Figure 1:**
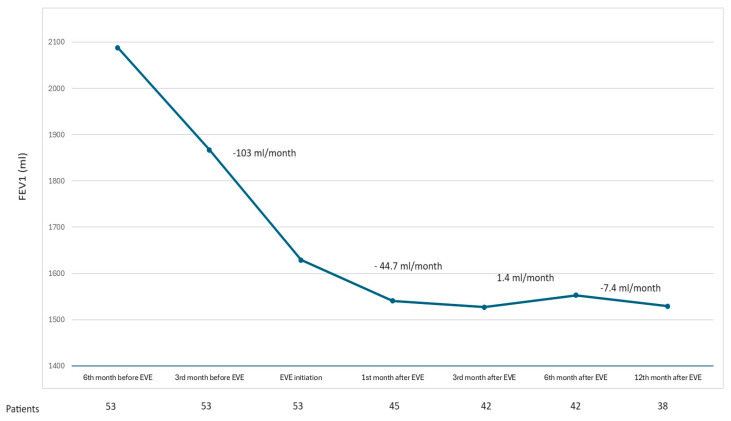
Slope of FEV_1_ variation before and after everolimus treatment.

The mean FEV_1_ values at six months, three months, and one month before the onset of everolimus therapy were 2088.1 (714.1) mL, 1867.4 (710.3) mL, and 1629.2 (556) mL, respectively. After one, three, six, and twelve months since the initiation of the imTOR therapy, the mean FEV_1_ values were 1541 (498) mL, 1527.8 (570.1) mL, 1553.1 (605.3) mL, and 1529.7 (622.3) mL, respectively. The FEV_1_ decline significantly improved after the initiation of everolimus from −102.7 (149.6) mL/month before starting everolimus to −44.7 (109.6) mL/month within the first three months; +1.4 (63.5) mL/month until the sixth month, and −7.4 (46.2) mL/month until the twelfth month. The differences in these slopes are statistically significant compared to the six months before the administration of the drug (*p* < 0.05) (Figure 1). The FEV_1_ improvement within the 12 months after everolimus was significantly higher in patients with BOS 0p or BOS 1 (a median FEV_1_ decline of −1.5 (−8.3–8.3) mL/month), compared to patients with BOS 2 or BOS 3 (−31.3 (−40–−5.8) mL/month) (*p* < 0.05).

### 3.2. Lipid Profile, Renal Function, and Immunosuppressive Dosage

Serum cholesterol levels at baseline and at 1, 3, and 6 months after the treatment change were 187 (35.3) mg/dL, 230 (59.7) mg/dL, 212.3 (47.9) mg/dL, and 207.1 (36.6) mg/dL, respectively (*p* < 0.05). Accordingly, the differences among triglycerides levels were statistically significant at the same time points [143.4 (71.1) mg/dL, 197.3 (104) mg/dL, 192.9 (100.3) mg/dL, and 188.3 (85.4) mg/dL, respectively] (*p* < 0.05). GF remained unchanged after everolimus treatment [59.1 (17.5) mL/min per 1.73 m^2^ at baseline and 60.9 (19.6) mL/min per 1.73 m^2^, 57.7 (20.5) mL/min per 1.73 m^2^, and 57.3 (17.8) mL/min per 1.73 m^2^ at 1, 3, and 6 months, respectively] (Table 2).

### 3.3. Immunosuppression

The mean everolimus levels at 1, 3, and 6 months were 5.1 (1.8) ng/mL, 5.9 (1.9) ng/mL, and 5.4 (1.8) ng/mL. As expected, tacrolimus serum levels decreased significantly after starting everolimus therapy (9.1 (3.5) ng/mL at baseline and 7.3 (3) ng/mL, 6.4 (2.2) ng/mL, and 5.7 (1.7) ng/mL, at 1, 3, and 6 months, respectively) (*p* < 0.05). (Table 2). In addition to tacrolimus and corticosteroids, the majority of the patients were receiving MMF or MPA before the onset of everolimus therapy. At baseline, 70.2% of them received a dose of ≥750 mg/day of MMF or ≥540 mg/day of MPA, 15.8% received 500–750 mg/day of MMF or 360–540 mg/day of MPA, and 1.8% received < 500 mg/day of MMF or <360 mg/day of MPA. After three months of everolimus treatment, 48.9% of the patients were not taking any antimetabolite drugs, while 8.9% continued to take 500–750 mg/day of MMF or 360–540 mg/day of MPA, and 42.2% received ≥ 750 mg/day of MMF or ≥540 mg/day of MPA. Regardless of the different azithromycin treatments between the two centers, all patients were taking this drug before the onset of everolimus treatment, and it was continued thereafter.

### 3.4. Adverse Events and Mortality

Everolimus was withdrawn in 22 (38.6%) patients. The median time to withdrawal was 14.1 (5.5–25.1) months. The primary reasons for the removal of the therapy were edema (15.8%), CLAD progression (10.5%), and leukopenia (5.3%).

The median transplantation survival time was 78 (39.9–142.9) months. No statistically significant differences were found regarding everolimus withdrawal (log-rank *p* = 0.9). The most relevant death causes were CLAD (38.6%), COVID-19 pneumonia (10.5%), malignancy (7%), and fungal infection (3.5%).

## 4. Discussion

Our study highlights how everolimus treatment improved the loss of lung function and therefore slowed the progression of CLAD in a cohort of 57 LT patients. It provides relevant clinical data by showing a beneficial effect on the progression of the leading cause of death in patients with LT. Although the number of patients is small, it delivers suitable information in a field with a great scarcity of studies and in which no therapy has proven effective. According to CLAD pathophysiology, it is reasonable to expect that the sooner the treatment is started, the more effective it will be. Delaying its initiation may lead to irreversible damage to lung function. In our series, the majority of the patients were in an early phase of CLAD (BOS 0p and BOS 1). The benefit was significantly higher in patients with a mild CLAD stage (BOS 0p and BOS 1) than those with a moderate or severe stage (BOS 2 and BOS 3). This finding enforces the concept of treating these patients in an early stage in order to obtain more significant benefits.

Everolimus therapy is a versatile treatment with multiple applications, including its use as a CNI-sparing agent in cases of renal insufficiency or malignancy. Previous research on CLAD has primarily focused on the prevention of this condition by administering everolimus early after transplantation. However, the clinical efficacy of the imTOR in treating established CLAD remains limited and requires further investigation. Previous studies, which were also retrospective with few patients, showed a stabilization in the FEV_1_ after everolimus therapy in patients with CLAD [19,21,25]. However, they did not provide information on the deterioration rate in the months previous to and following the onset of everolimus. Additionally, they did not provide data on the CLAD stage in which the drug had been introduced. To this end, several clinical trials have been conducted to assess the clinical effectiveness of everolimus therapy. Snell et al. compared everolimus with azathioprine (added to CNI and corticosteroids in both groups) and described a smaller decrease in the FEV_1_ in the twelve months after starting treatment as well as a lower number of acute rejections in LT with everolimus, although none of these patients had established CLAD before participating in the trial [12]. Similarly, a European and Australian group compared everolimus to sodium mycophenolate associated with cyclosporine and corticosteroids and found no differences in the incidence of BOS. Again, this trial was designed to assess the prevention of CLAD and not its effect on an established condition [13]. Recently, a German trial was carried out in order to evaluate the superiority of everolimus with low CNI exposure in a quadruple immunosuppression regimen in terms of renal function. It demonstrated a lower prevalence of CLAD and a higher CLAD-free survival time in the quadruple therapy group. However, the difference was not statistically significant [17].

In our cohort, the use of everolimus did not demonstrate differences in renal function after one year of treatment, maintaining similar creatinine and renal filtrate levels. Regarding this matter, the published studies reveal a disparity of information. Gullestad et al. [26] showed long-term renal improvement by combining de novo everolimus with reduced doses of CNI in cardiac transplantation, but this benefit was not achieved in LT. Gottlieb et al. demonstrated a benefit in one-year renal function in LT in patients with quadruple therapy with reduced exposure to CNI. However, this benefit was not maintained after five years [17]. There needs to be more scientific evidence for the appropriate ranges for these combined drugs. Previous studies [27] proposed a trough range between 3 and 12 ng/mL, which is a broad range. Thus, based on previous experience, the Spanish consensus [18] proposes maintaining narrower ranges between 3 and 8 ng/mL. Since the patients in this study were diagnosed with CLAD, the protocols of both centers aimed to reach everolimus trough levels between 5 and 8 ng/mL and tacrolimus trough levels between 5 and 8 ng/mL. Despite differences in the initial azithromycin protocol between the two centers, all patients were taking this drug upon starting everolimus. The steroid dose was unchanged and, in some cases, according to the criteria of the responsible physician, a fourth immunosuppressive line with mycophenolate was maintained. However, as observed in the results, the average trough levels achieved are somewhat lower than recommended in the protocol, probably because of the frequent side effects, which forced us to reduce the levels. Despite this, a clinical benefit is observed as the loss of lung function was slowed without damaging renal function.

Tolerance is a crucial factor to consider when administering this drug. There are numerous adverse events that have been reported, such as oral ulcers, edema, proteinuria, and cytopenia. Due to the severity of these events, it is often necessary to discontinue treatment. Caution should be exercised when administering this drug to prevent the onset of these adverse events. Similar to other published series, the patients in this study frequently presented side effects [13,15,17]. Although most patients were treated to mitigate the well-known side effect of a worsening lipid profile, it still significantly deteriorated. However, this was not a reason for discontinuing the medication. The percentage of drug withdrawals due to adverse events was higher than in previously published data, with peripheral edema and leukopenia being the primary reasons for withdrawal. It is worth noting that they were described during a notably longer follow-up period than other published studies. These withdrawals emerged over time, indicating that although the drug may be tolerated initially, some patients become intolerant as time passes. It is important to highlight that during the course of the study, 10.5% of the participants withdrew from the treatment due to a lack of efficacy and the progression of CLAD. Therefore, these withdrawals should not be deemed side effects of the treatment.

CLAD poses a significant challenge to long-term survival in lung transplant recipients. To address this issue, there is a pressing need to conduct more clinical trials to assess the prevention and treatment of CLAD. However, the high heterogeneity of this condition and the diverse causes that can worsen lung function, as well as the different CLAD phenotypes, make it challenging to design clinical trials. Therefore, a deeper understanding of the pathophysiology and more accurate patient phenotyping are critical for addressing this issue.

Our work has some inherent limitations due to its retrospective nature. Not documenting all side effects but only those that conditioned the withdrawal of the drug probably resulted in an underestimation of toxicity. The absence of a control group is attributable to the fact that our protocol indicates initiating everolimus if CLAD is diagnosed and the patient is eligible to take the drug. Consequently, other patients diagnosed with CLAD who are not taking everolimus differ in characteristics from the studied group. However, this study comprises the most extensive series of patients with LT and established CLAD and the longest follow-up period analyzed for patients under everolimus treatment. It is also the only study that not only measures FEV_1_ changes after the onset of everolimus but also analyzes the slope of functional loss before and after its initiation.

## 5. Conclusions

Our research revealed that the administration of everolimus in treating CLAD can lead to a significant reduction in the loss of lung function, particularly in patients with early-stage CLAD. Unfortunately, a sizable proportion of patients had to discontinue the treatment owing to side effects. To enhance the long-term prognosis of lung transplant recipients, we must gain a more comprehensive understanding of the pathophysiology of CLAD and identify the patients who are most likely to benefit from this therapy. This will enable us to conduct more extensive studies that can yield data to evaluate the effectiveness of everolimus and other medications.

## Figures and Tables

**Table 1:** Baseline clinical and demographic characteristics of lung transplant patients (N = 57) in whom everolimus for CLAD was initiated.

Baseline Characteristics	
Gender, male	38 (67%)
Age at transplantation (years), median (IQR)	55.8 (51.2–61.5)
Indication for lung transplantation	
Chronic obstructive pulmonary disease	29 (51%)
Interstitial lung disease	17 (30%)
Pulmonary arterial hypertension	5 (9%)
Other causes	6 (10%)
Type of transplantation	
Bilateral	36 (63%)
Basiliximab induction	26 (45.6%)
CMV mismatch	4 (7%)
Baseline BOS stage	
BOS 0p	18 (31.6%)
BOS 1	14 (24.6%)
BOS 2	13 (22.8%)
BOS 3	6 (10.5%)
Months from transplantation to everolimus	27.1 (16–38.8)
Months in treatment with everolimus	21.6 (6.1–73.7)
Follow up, months	45.5 (11.1–92.1)
Drug withdrawal	22 (38.6%)
Months to withdrawal	14.1 (5.5–25.1)
Cause of withdrawal	
Edema	9 (15.8%)
CLAD progression	6 (10.5%)
Leukopenia	3 (5.3%)
Proteinuria	1 (1.8%)
Diarrhea	1 (1.8%)
Infection	1 (1.8%)
Thrombotic microangiopathy	1 (1.8%)
Others	8 (14%)

**Table 2:** Lipid profile, renal function, and immunosuppression during the first 6 months with everolimus (N = 57).

	Day 0	Month 1	Month 3	Month 6	*p* Value
Cholesterol (mg/dL)	187 (35.3)	229.9 (59.7) *	212.3 (47.9) *	207.1 (36.6) *	<0.05
Triglycerides (mg/dL)	143.4 (71.1)	197.3 (104) *	192.9 (100.3) *	188.3 (85.4) *	<0.05
Creatinine (mg/dL)	1.2 (0.4)	1.2 (0.4)	1.3 (0.5)	1.2 (0.4)	ns
GF (mL/min/1.73 m^2^)	59.1 (17.5)	60.9 (19.6)	57.7 (20.5)	57.3 (17.8)	ns
Everolimus (ng/mL)		5.1 (1.8)	5.9 (1.9)	5.4 (1.8)	ns
Tacrolimus (ng/mL)	9.1 (3.5)	7.3 (3)	6.4 (2.2)	5.7 (1.7)	<0.05

* *p* < 0.05 compared to day 0. ns: non statistic difference.

## Data Availability

Data is archived and can be provided by the authors.
